# PD-1/PD-L1抑制剂在晚期非小细胞肺癌中的治疗进展

**DOI:** 10.3779/j.issn.1009-3419.2017.02.09

**Published:** 2017-02-20

**Authors:** 战宇 潘

**Affiliations:** 1 300060 天津，天津医科大学肿瘤医院，国家肿瘤临床医学研究中心，天津市“肿瘤防治”重点实验室，天津市恶性肿瘤临床医学研究中心，天津市肿瘤免疫与生物治疗重点实验室，中西医结合科 Department of Synergistic Chinese and Western Medicine, Tianjin Medical University Cancer Institute and Hospital; National Clinical Research Center of Cancer; Key Laboratory of Cancer Prevention and Therapy, Tianjin; Tianjin's Clinical Research Center for Cancer; Key Laboratory of Cancer Immunology and Biotherapy, Tianjin 300060, China; 2 300060 天津，天津医科大学肿瘤医院，国家肿瘤临床医学研究中心，天津市“肿瘤防治”重点实验室，天津市恶性肿瘤临床医学研究中心，天津市肿瘤免疫与生物治疗重点实验室，免疫室 Department of Immunology, Tianjin Medical University Cancer Institute and Hospital; National Clinical Research Center of Cancer; Key Laboratory of Cancer Prevention and Therapy, Tianjin; Tianjin's Clinical Research Center for Cancer; Key Laboratory of Cancer Immunology and Biotherapy, Tianjin 300060, China

**Keywords:** 肺肿瘤, 程序性死亡受体1, 程序性死亡配体1, Lung neoplasms, Programmed death 1, Programmed death 1

## Abstract

程序性死亡受体1（programmed death 1, PD-1）抑制剂Pembrolizumab进入一线正式标志着免疫检查点抑制剂在晚期非小细胞肺癌（non-small cell lung cancer, NSCLC）的治疗体系中占据了重要地位。临床试验结果证实PD-1/程序性死亡配体1（programmed death ligand 1, PD-L1）抑制剂在晚期NSCLC的一线、二线和多药耐药后治疗的疗效均要优于传统的化疗。一线使用Pembrolizumab联合化疗的客观有效率（objective response rate, ORR）最高可达80%；单药Pembrolizumab的无疾病进展时间（progression-free survival, PFS）接近1年（10.3个月），死亡风险比含铂双药化疗下降40%。单药Pembrolizumab、Nivolumab和Atezolizumab用于二线的疗效同样突出，总生存时间（overall survival, OS）可至1年左右。PD-L1的表达是PD-1/PD-L1抑制剂疗效的预测因子，在晚期NSCLC中阳性（≥1%）的比例约为60%左右，组织类型间差异不大，但是目前并无检测的金标准。

## Abstract (en)

Pembrolizumab, an inhibitor target programmed death 1 (PD-1), was approved into the first line therapy in advanced non-small cell lung cancer (NSCLC). It was a milestone that immune checkpoints drugs have played an important role in the treatment system of NSCLC. The results of clinical trials revealed the superiority of PD-1/programmed death ligand 1 (PD-L1) inhibitors compared with chemotherapy in first-line, second-line and multidrug resistance phase therapy. Objective response rate (ORR) was up to 80% with pembrolizumab plus chemotherapy, and progression-free survival (PFS) with single pembrolizumab in first line was nearly 1 year (10.3 months), the hazard ratio for death fell by 40%. Overall survival (OS) was more or less 1 year with single drug pembrolizumab, nivolumab and atezolizumab for second line therapy. PD-L1 expression was a predictor of PD-1/PD-L1 inhibitors. The positive rate of PD-L1 (more than 1%) in advanced NSCLC was about 60% with little difference between the tissue types. However, there was no gold standard test of PD-L1 expression.

2016年10月24日美国食品药品管理局（Food and Drug Administration, FDA）正式批准程序性死亡受体1（programmed death 1, PD-1）抑制剂Pembrolizumab用于一线治疗晚期非小细胞肺癌（non-small cell lung cancer, NSCLC）。随后，美国国立综合癌症网络（National Comprehensive Cancer Network, NCCN）指南于10月26日发布的最新版本也对此进行了更新，推荐对于程序性死亡配体1（programmed death ligand 1, PD-L1）高表达（Tumor Proportion Score, TPS; ≥50%）的晚期NSCLC患者可一线使用Pembrolizumab。这是免疫检查点抑制剂在晚期NSCLC治疗中的重大突破，标志着免疫治疗在晚期NSCLC的一线治疗中占据了一席之地。除Pembrolizumab外，另外一种PD-1抑制剂Nivolumab和一种PD-L1抑制剂Atezolizumab在最新的NSCLC NCCN指南中也被推荐为二线治疗。本文将就以上3种PD-1/PD-L1抑制剂在晚期NSCLC中的治疗进展做一综述。

## 1 PD-1/PD-L1信号通路与NSCLC

PD-1和PD-L1是一对免疫抑制性因子，PD1/PD-L1通路的激活可导致免疫抑制性肿瘤微环境形成，使肿瘤细胞逃避机体免疫监视和杀伤^[1]^。PD-1是免疫球蛋白B7-CD28家族成员之一，主要在免疫细胞（活化的CD4^+^T细胞、CD8^+^T细胞、B细胞、自然杀伤T细胞、单核细胞和树突状细胞）中表达^[2]^。PD-L1是PD-1的主要配体，在很多恶性肿瘤细胞中过表达，其中就包括NSCLC^[3-4]^，乳腺癌^[5]^和黑色素瘤^[6]^等。除肿瘤细胞外，PD-L1还在肿瘤浸润性树突状细胞、肿瘤浸润性淋巴细胞、肿瘤浸润性巨噬细胞中表达^[7, 8]^。PD-1和PD-L1结合后可抑制CD4^+^T细胞、CD8^+^T细胞的增殖和活力，这在正常人表现为减少免疫反应对周围组织的损伤，避免发生自身免疫性疾病^[2, 9]^，而在肿瘤患者体内则可使肿瘤局部微环境的T细胞免疫杀伤作用降低，从而导致肿瘤免疫逃逸，促进肿瘤生长^[1]^。因此，阻断PD-1和PD-L1结合在理论上可以恢复肿瘤微环境内T细胞的免疫杀伤作用，从而达到抑制肿瘤的目的。目前针对PD-1/PD-L1的药物已在黑色素瘤、肾癌、头颈部鳞癌和NSCLC等多种恶性肿瘤中被批准应用。

多项临床研究证实PD-1/PD-L1抑制剂在晚期NSCLC中有卓越疗效，也发现PD-L1表达水平与疗效密切相关，是直接的疗效预测因子。因此，了解PD-L1在晚期NSCLC中的表达比例及其与组织类型是否相关非常重要。KEYNOTE多项研究均未限定组织类型，但以非鳞癌居多；在KEYNOTE-001研究^[10]^中接受PD-L1检测的患者824例，其中501例（60.8%）≥1%，191例（23.2%）≥50%；KEYNOTE-010研究^[11]^纳入2, 222例患者，其中1, 475例（66%）≥1%，633例（28%）≥50%；KEYNOTE-024^[12]^研究检测了1, 653例患者，其中≥50%的比例是30.2%。而CheckMate-063^[13]^和CheckMate-017^[14]^这两项研究入组患者均为鳞癌，前者结果显示PD-L1≥5%的比例为33%（25/76）；后者显示PD-L1≥10%的比例为30.7%（69/225），≥1%的比例为52.9%（119/225）。OAK研究^[15]^同样未限定组织类型，结果显示PD-L1≥50%的比例为16%（137/850），≥1%的比例为54%（463/850）。总结以上数据可以发现，PD-L1在晚期NSCLC中呈高表达状态，其中≥1%的患者比例为52.9%-66%；≥50%的患者比例是16%-30.2%，鳞癌与非鳞癌未见明显差异。

## 2 PD-1/PD-L1抑制剂在晚期NSCLC中的临床应用

目前，已被批准应用于晚期NSCLC的PD-1/PD-L1通路的免疫检查点抑制剂主要有三种：针对PD-1的单克隆抗体：Pembrolizumab（MK-3475）和Nivolumab（BMS-936558）；针对PD-L1的单克隆抗体：Atezolizumab（MPDL-3280A）。

### 2.1 Pembrolizumab（KEYNOTE系列试验）

KEYNOTE-001试验^[10]^是首先启动的Ⅰ期临床研究，结果发现在晚期NSCLC患者中，Pembrolizumab单药的客观有效率（objective response rate, ORR）和有效持续时间（duration of response, DOR）是19.4%和12.5个月，中位无疾病进展时间（progression-free survival, PFS）和总生存时间（overall survival, OS）分别为3.7个月和12个月。亚组分析显示例初治患者疗效要好于经治患者，ORR提高了6.8%（24.8%和18%）；DOR、PFS和OS也均有1倍左右的延长。除此之外还发现PD-L1高表达（TPS≥50%）组疗效更为显著，ORR达到45.2%，PFS是6.3个月，其中的初治患者PFS更是达到1年以上（12.5个月）。基于此项结果，美国FDA在2015年10月2日通过了“Pembrolizumab用于治疗PD-L1阳性且在含铂化疗期间或之后发生疾病进展的转移性NSCLC患者”的快速审批。

此后，3项前瞻性的随机对照临床研究探讨了Pembrolizumab在晚期NSCLC中的二线（KEYNOTE-010研究）、一线（KEYNOTE-024研究）以及与化疗联合应用于一线（KEYNOTE-021研究）的疗效优劣。

KEYNOTE-010^[11]^是第1项将Pembrolizumab与化疗进行比较的随机对照研究，入组患者要求为一线治疗后进展且PD-L1阳性（≥1%）的晚期NSCLC。1, 034例患者按1:1:1的比例随机分配至Pembrolizumab低剂量组（2 mg/kg，每3周1次）和高剂量组（10 mg/kg，每3周1次）或多西他赛组。结果显示在总体人群中，3组患者中位OS分别为10.4个月、12.7个月和8.5个月，Pembrolizumab低、高剂量组与化疗组比较均有统计学差异（*P* < 0.001）；不过PFS数据相当，分别为3.9个月、4.0个月和4.0个月。在PD-L1高表达（TPS≥50%）的亚组中，Pembrolizumab低剂量和高剂量组的中位OS分别为14.9个月和17.3个月，中位PFS为5.0个月和5.2个月，与化疗组（8.2个月和4.1个月）相比均有统计学差异。该研究首次前瞻性将PD-L1表达来预测Pembrolizumab疗效，也最终证实PD-L1表达水平可作为Pembrolizumab疗效预测的生物标记物；同时也确定了最佳剂量为2 mg/kg。

与标准含铂双药进行一线比较的临床研究是KEYNOTE-024，其结果在前不久结束的欧洲肿瘤年会（European Society for Medical Oncology, ESMO）上披露，入组患者要求PD-L1高表达（TPS≥50%）^[12]^。结果显示与化疗比较，Pembrolizumab组PFS延长了4.3个月（10.3个月*vs* 6.0个月，HR=0.50，*P* < 0.001）；因随访时间较短，中位OS的数据尚未得到，但是与化疗相比，Pembrolizumab治疗组死亡风险下降了40%（HR=0.60, *P*=0.005）。在ORR方面，Pembrolizumab同样优于化疗（44.8% *vs* 27.8%），并且DOR更长。此项研究结果首次证实了Pembrolizumab单药一线治疗晚期NSCLC优于标准的含铂双药化疗，也直接促使了美国FDA和NCCN指南将Pembrolizumab推荐用于晚期NSCLC的一线治疗。

KEYNOTE-021研究^[16]^尝试Pembrolizumab与化疗联用一线治疗晚期NSCLC，组织类型限定为腺癌，化疗方案选择培美曲塞联合卡铂。结果发现首要观察终点ORR在联合治疗组为55%，化疗组仅为29%；中位PFS分别为13个月和8.9个月（HR=0.53, *P*=0.01）。按PD-L1的表达水平分层发现在PD-L1 < 1%、1%-49%和≥50%的患者中，ORR在联合治疗组分别为57%、26%和80%，化疗组分别为13%、39%和35%，PD-L1高表达组（≥50%）疗效最优。与前面的KEYNOTE-024研究结果相比，Pembrolizumab联合化疗与单药相比，PFS延长了2.6个月（13个月和10.4个月），ORR提高了10.2%（55%和44.8%），在PD-L1高表达（TPS≥50%）的亚组中，联合治疗组ORR高达80%。但需要注意的是，该项临床研究入组患者人数较少，仅有123例（联合治疗组60例，化疗组63例）。如果想进一步了解Pembrolizumab联合化疗与Pembrolizumab单药比较的安全性和疗效优劣，有必要开展“头对头”的随机对照临床研究。

从以上几项临床研究可以看出，Pembrolizumab治疗晚期NSCLC疗效确切，无论是一线的初治患者，还是经历过化疗后的二线及以后的复治患者均可获益，尤其是对于PD-L1高表达（TPS≥50%）的患者。此外，由于在KEYNOTE-001临床研究中注意到：在初治患者中，PD-L1中度表达（1%-49%）组应用Pembrolizumab治疗的PFS和OS均优于PD-L1 < 1%组，因此KEYNOTE-042临床研究设计针对PD-L1≥1%的晚期NSCLC患者比较Pembrolizumab与含铂双药化疗在一线治疗中的优劣，非常期待此项研究结论能给我们带来新的提示。

### 2.2 Nivolumab（CheckMate系列试验）

1项Ⅰ期、剂量爬坡研究^[17, 18]^将Nivolumab分成3个剂量组：1 mg/kg、3 mg/kg和10 mg/kg，纳入了129例晚期NSCLC患者，其中三线及以上的治疗比例为54.3%。结果发现全部患者中位OS为9.9个月，3 mg/kg剂量组疗效最佳，中位OS达到14.9个月，而1 mg/kg和10 mg/kg组的中位OS均为9.2个月。鳞癌和非鳞癌数据相当。CheckMate-063同样是一项评估单药的单臂Ⅱ期临床研究^[13]^，入组患者被限定为晚期耐药鳞癌，其中大部分患者也处于三线及以后治疗的状态。结果显示Nivolumab单药治疗的ORR为14.5%，中位OS是8.2个月，1年生存率达到40.8%。该生存数据远远超过既往关于晚期耐药鳞癌研究结果，因此Nivolumab也成为第一个被美国FDA批准应用于晚期鳞癌的免疫检查点抑制剂（2015年3月4日）。

随后开展的两项Ⅲ期随机临床对照研究CheckMate-017^[14]^和CheckMate-057^[19]^针对既往含铂方案一线治疗进展的晚期NSCLC进行了关于Nivolumab的二线探索。患者按1:1随机分组接受Nivolumab或多西他赛治疗，主要研究终点是OS。不同之处在于入组患者的组织类型前者限定为鳞癌，后者为非鳞癌。CheckMate-017 ^[14]^共纳入272例晚期鳞癌，结果显示Nivolumab组与多西他赛组的中位OS分别为9.2个月和6.0个月，Nivolumab组死亡风险下降了41%（HR=0.59, *P* < 0.001）。两组ORR分别为20%和9%（*P*=0.008）；中位PFS分别为3.5个月和2.8个月（HR=0.62, *P* < 0.001）。研究者将PD-L1的表达水平按1%、5%和10%进行分层后对比发现PD-L1的表达不能作为Nivolumab的生物标记，与疗效和预后均无明显关系。CheckMate-057^[19]^共入组582例患者，结果发现两组的中位OS分别为12.2个月和9.4个月（HR=0.73, *P*=0.002）；ORR分别为19%和12%（*P*=0.02）；但中位PFS无统计学差异。不过，与CheckMate-017不同的是，本研究发现随着PD-L1表达水平的增加（按1%、5%和10%进行递增），Nivolumab的疗效亦有所提高，但未提供具体数值。基于这些结果，美国FDA更改Nivolumab的适应症为不限组织类型的晚期NSCLC二线治疗（2015年10月）。

还有两项Ⅰ期临床研究探索了Nivolumab一线治疗晚期NSCLC的安全性和疗效。其中CheckMate-012研究^[20]^观察到Nivolumab单药治疗晚期NSCLC的ORR是23%，中位PFS和OS分别为3.6个月和19.4个月，在PD-L1≥1%的患者中，ORR有所提高，达到28%。另一项临床研究^[21]^一线采用Nivolumab联合化疗，结果发现不良反应发生率会明显提高；与其他化疗方案相比，紫杉醇/卡铂与Nivolumab联合应用疗效更优。但是这两项Ⅰ期研究的患者人数都比较少，分别是52例和56例。Nivolumab未来能否进入到一线治疗需要大规模的随机对照研究的结果来证实。

### 2.3 PD-L1抗体-Atezolizumab

早期的非随机对照临床研究已证实Atezolizumab在晚期NSCLC的疗效确切、不良反应发生率低^[22]^。2013年8月开始的POPLAR研究^[23]^是一项随机对照的Ⅱ期临床研究，主要目的是在评价Atezolizumab二线应用的有效性和安全性。287例患者按1:1随机分配至Atezolizumab组或多西他赛组。结果显示首要研究终点OS在Atezolizumab组和多西他赛组分别为12.6个月和9.7个月（HR=0.73, *P*=0.04），PFS无明显差异（2.7个月和3.0个月，HR=0.94）。DOR在Atezolizumab组要明显优于多西他赛组（14.3个月和7.2个月）。该研究分别在肿瘤细胞（tumor cell, TC）和肿瘤浸润免疫细胞（tumor-infiltrating immune cells, TIC）中检测了PD-L1的表达，亚组分析显示，PD-L1≥1%的患者接受Atezolizumab治疗后中位OS比化疗延长了6.3个月（15.5个月*vs* 9.2个月，HR=0.59，*P*=0.005）；而对于PD-L1 < 1%的患者，两组OS相当，均为9.7个月。在此基础上的Ⅲ期临床研究-“OAK”^[15]^与POPLAR研究目的相同，即二线应用Atezolizumab与多西他赛进行比较。其结果在2016年的ESMO会议上进行了汇报，Atezolizumab治疗组的中位OS比化疗组延长了4.2个月（13.8个月*vs* 9.6个月，HR=0.74，*P*=0.000, 4），无论PD-L1表达与否，Atezolizumab与化疗比均可获益，组织类型也并未影响Atezolizumab的疗效。基于此两项研究结果，2016年10月19日美国FDA批准Atezolizumab二线治疗晚期NSCLC。

## 3 PD-1/PD-L1抑制剂的不良反应及应用禁忌

PD-1/PD-L1抑制剂作为一类新的治疗药物，其不良反应也不同于传统化疗药物或分子靶向药物，目前研究认为其不良反应是非特异性免疫活性所导致。免疫相关不良反应可以累及任何器官或系统，但主要受累的有皮肤、胃肠道、肝脏、肺及内分泌系统等，表现为皮疹、结肠炎、肝炎、肺炎和甲状腺功能的亢进或减退。不过这些不良反应多是1级-2级，3级以上的相关不良反应少见，多在5%以下；三种药物的不良反应发生率也基本相当，严重不良事件发生率都明显低于化疗。鉴于免疫相关的不良反应，对于合并有自身免疫性疾病（需要免疫抑制剂治疗）、有症状的间质性肺疾病、需要类固醇激素治疗的肺炎和伴有全身性免疫抑制的患者是不建议使用PD-1/PD-L1抑制剂进行治疗的。

## 4 PD-1/PD-L1抑制剂临床应用的一些问题

以上所有临床研究都在肿瘤细胞中进行了PD-L1的表达，并且发现PD-L1的表达情况对药物的疗效有预测作用，但是依然有很多局限和不足。①目前并无PD-L1检测的“金标准”，正在使用的试剂盒及抗体有三种（Dako 22C3, Dako 28-8, VentanaSP14），前两者的阳性定义标准是TC表面PD-L1表达 > 1%，而后者是在TC和TIC两者均进行检测，两者表达均 < 1%时设为阴性，其他情况均视为阳性。②一线使用Pembrolizumab的标准是PD-L1的TPS≥50%，但在二线使用PD-1/PD-L1抑制剂的标准并不统一，Pembrolizumab要求PD-L1≥1%，而Nivolumab和Atezolizumab是无需确认PD-L1是否表达。③由于肿瘤的异质性，晚期肺癌的活检标本会不会导致“假阳性”和“假阴性”的结果也需要注意，随着血液检测的技术进步，能否找到替代的血液检测指标（诸如*PD*-*1*和*PD*-*L1*基因状态、肿瘤的突变负荷等）同样重要。④免疫检查点抑制剂的一线适应症是无驱动基因突变的晚期NSCLC，在几项二线的临床研究^[11, 15, 19]^纳入了少部分合并有驱动基因突变的患者（*EGFR*、*KRAS*等），不过在亚组分析中发现合并有EGFR驱动基因突变的患者不能从PD-1/PD-L1抑制剂的治疗中获益，甚至疗效不如化疗。最后，目前一线治疗仅有Pembrolizumab被批准，但是对于铂类耐药后的二线治疗，Pembrolizumab、Nivolumab和Atezolizumab均可作为被选择的药物，如何为患者选择性价比最高的药物也需要临床医生慎重考虑。

## 5 总结

综上所述，针对PD-1/PD-L1的免疫检查点抑制剂在晚期NSCLC的治疗中显示出了令人瞩目的卓越疗效。对于无驱动基因突变的晚期NSCLC，免疫检查点抑制剂无论在一线、二线还是二线以后的治疗中均显示出了超过化疗的疗效。而且，PD-L1≥1%的患者比例在晚期NSCLC可达60%左右，超过了驱动基因突变的患者人群，意味着多数患者可以接受免疫检查点抑制剂的治疗并从中获益。目前，最重要的是找到最佳的可预测疗效的生物标记生物标记物，来指导晚期NSCLC的临床“精准”治疗。

## References

[1] Pardoll DM (2012). The blockade of immune checkpoints in cancer immunotherapy. Nat Rev Cancer.

[2] Keir ME, Butte MJ, Freeman GJ (2008). PD-1 and its ligands in tolerance and immunity. Annu Rev Immunol.

[3] Velcheti V, Schalper KA, Carvajal DE (2014). Programmed death ligand-1 expression in non-small cell lung cancer. Lab Invest.

[4] Boland JM, Kwon ED, Harrington SM (2013). Tumor B7-H1 and B7-H3 expression in squamous cell carcinoma of the lung. Clin Lung Cancer.

[5] Bae SB, Cho HD, Oh MH (2016). Expression of programmed death receptor ligand 1 with high tumor-infiltrating lymphocytes is associated with better prognosis in breast cancer. J Breast Cancer.

[6] Spranger S, Spaapen RM, Zha Y (2013). Up-regulation of PD-L1, IDO, and T(regs) in the melanoma tumor microenvironment is driven by CD8(+) T cells. Sci Transl Med.

[7] Mu CY, Huang JA, Chen Y (2011). High expression of PD-L1 in lung cancer may contribute to poor prognosis and tumor cells immune escape through suppressing tumor infiltrating dendritic cells maturation. Med Oncol.

[8] Taube JM, Klein A, Brahmer JR (2014). Association of PD-1, PD-1 ligands, and other features of the tumor immune microenvironment with response to anti-PD-1 therapy. Clin Cancer Res.

[9] Keir ME, Liang SC, Guleria I (2006). Tissue expression of PD-L1 mediates peripheral T cell tolerance. J Exp Med.

[10] Garon EB, Rizvi NA, Hui R (2015). Pembrolizumab for the treatment of non-small-cell lung cancer. N Engl J Med.

[11] Herbst RS, Baas P, Kim DW (2016). Pembrolizumab versus docetaxel for previously treated, PD-L1-positive, advanced non-small-cell lung cancer (KEYNOTE-010): a randomised controlled trial. Lancet.

[12] Reck M, Rodriguez-Abreu D, Robinson AG (2016). Pembrolizumab versus chemotherapy for PD-L1-positive non-small-cell lung cancer. N Engl J Med.

[13] Rizvi NA, Mazieres J, Planchard D (2015). Activity and safety of nivolumab, an anti-PD-1 immune checkpoint inhibitor, for patients with advanced, refractory squamous non-small-cell lung cancer (CheckMate 063): a phase 2, single-arm trial. Lancet Oncol.

[14] Brahmer J, Reckamp KL, Baas P (2015). Nivolumab versus docetaxel in advanced squamous-cell non-small-cell lung cancer. N Engl J Med.

[15] Rittmeyer A, Barlesi F, Waterkamp D (2017). Atezolizumab versus docetaxel in patients with previously treated non-small-cell lung cancer (OAK): a phase 3, open-label, multicentre randomised controlled trial. Lancet.

[16] Langer CJ, Gadgeel SM, Borghaei H (2016). Carboplatin and pemetrexed with or without pembrolizumab for advanced, non-squamous non-small-cell lung cancer: a randomised, phase 2 cohort of the open-label KEYNOTE-021 study. Lancet Oncol.

[17] Topalian SL, Hodi FS, Brahmer JR (2012). Safety, activity, and immune correlates of anti-PD-1 antibody in cancer. N Engl J Med.

[18] Gettinger SN, Horn L, Gandhi L (2015). Overall survival and long-term safety of nivolumab (Anti-Programmed Death 1 Antibody, BMS-936558, ONO-4538) in patients with previously treated advanced non-small-cell lung cancer. J Clin Oncol.

[19] Borghaei H, Paz-Ares L, Horn L (2015). Nivolumab versus docetaxel in advanced nonsquamous non-small-cell lung cancer. N Engl J Med.

[20] Gettinger S, Rizvi NA, Chow LQ (2016). Nivolumab monotherapy for first-line treatment of advanced non-small-cell lung cancer. J Clin Oncol.

[21] Rizvi NA, Hellmann MD, Brahmer JR (2016). Nivolumab in combination with platinum-based doublet chemotherapy for first-line treatment of advanced non-small-cell lung cancer. J Clin Oncol.

[22] Herbst RS, Soria JC, Kowanetz M (2014). Predictive correlates of response to the anti-PD-L1 antibody MPDL3280A in cancer patients. Nature.

[23] Fehrenbacher L, Spira A, Ballinger M (2016). Atezolizumab versus docetaxel for patients with previously treated non-small-cell lung cancer (POPLAR): a multicentre, open-label, phase 2 randomised controlled trial. Lancet.

